# A rare case of Weil’s disease with acute pancreatitis and acute kidney injury: focus on management – a case report

**DOI:** 10.1097/MS9.0000000000000387

**Published:** 2023-03-27

**Authors:** Reynaldo B. Hutajulu, Bramantono Bramantono, Musofa Rusli, Muhammad V. Arifijanto, Usman Hadi

**Affiliations:** a Department of Internal Medicine; b Tropical and Infectious Disease Research Group, Department of Internal Medicine, Faculty of Medicine, Universitas Airlangga – Dr. Soetomo General Academic Hospital, Surabaya, Indonesia

**Keywords:** acute kidney injury, acute pancreatitis, leptospirosis, Weil’s disease

## Abstract

The clinical spectrum of leptospirosis ranges from subclinical infection and anicteric fever to Weil’s disease, a severely fatal infection. Acute inflammation of the pancreas is a rare manifestation of Weil’s disease, and renal involvement is a hallmark in severe forms, leading to acute kidney injury (AKI) and representing a major risk factor for death. The aim of the case report was to present the clinical manifestations of Weil’s disease with acute pancreatitis and AKI and to highlight the management of its complications.

**Case presentation:**

A 22-year-old male patient presented to the hospital with a chief complaint of a persisting fever, abdominal pain, nausea and vomiting, decreased appetite, malaise, and urine and feces discoloration. The patient’s residence had flooded 2 weeks ago. Laboratory tests were performed and the patient was diagnosed with Weil’s disease with the complication of acute pancreatitis, AKI, hyperkalemia, hyponatremia, hypotonic hypovolemic, metabolic acidosis, and hypoalbuminemia.

**Clinical discussion:**

The patient was treated with intravenous (i.v.) ceftriaxone at a dose of 2×1 g, i.v. metoclopramide at a dose of 3×10 mg, i.v. calcium gluconate at a dose of 1 g followed by dextrose (D) 40% with insulin 2 IU for six times, avoided nephrotoxic drugs, and fluid balance was maintained at I=O+500 ml. The patient received hemodialysis due to refractory hyperkalemia. Posttreatment follow-up presented improvements in complaints and laboratory parameters.

**Conclusion:**

Management of severe leptospirosis or Weil’s disease with the complication of acute pancreatitis and AKI requires antibiotics and supportive therapy including adequate fluid resuscitation, proper and adequate nutrition, as well as the initiation of hemodialysis.

## Introduction

Highlights

- A rare case of Weil’s disease with acute pancreatitis and acute kidney injury is reported.
- The patient also had hyperkalemia, hyponatremia, hypovolemic, metabolic acidosis, and hypoalbuminemia.
- Appropriate antibiotics and supportive therapies avoided disease progression and improved the patient’s condition.

Leptospirosis is a zoonotic disease caused by bacterial pathogens of the genus *Leptospira* and most common in tropical and subtropical areas and is endemic to several places in Indonesia^1^. *Leptospira* serovars are naturally carried in wild and domestic animals, especially in the renal tubules of rodents, and the transmission occurs either directly through contact with infected animal urine or indirectly through contaminated water and soil^2^. The highest incidence of leptospirosis is in Africa, the Western Pacific, America, Southeast Asia, and Europe^2^. Several provinces in Indonesia are endemic for leptospirosis, and a study showed that the highest incidence was in Central Java and East Java^1^. A cohort study reported leptospirosis in 3.5% of hospitalized patients presenting acute febrile illness^1^.

The spectrum of leptospirosis is wide, ranging from subclinical infection, anicteric fever, to Weil’s disease, a severely fatal infection. Leptospirosis affects multiple organs, such as the central nervous system, eyes, heart, lungs, liver, and kidneys, including the pancreas^3^. Acute inflammation of the pancreas is a rare manifestation of Weil’s disease, and only two cases were reported in the *World Journal of Gastroenterology* 4. Acute pancreatitis is diagnosed based on clinical, laboratory, and radiological findings. A histopathologist is occasionally required to confirm the diagnosis. The management of acute pancreatitis in Weil’s disease includes anti-*Leptospira* antibiotics and supportive therapy to treat acute pancreatitis. Effective treatment is strongly advised since it lowers mortality^3^. There is no specific treatment for leptospiral-induced acute kidney injury (AKI), but it can be symptomatically managed. Initiation of dialysis in patients with AKI stage 3 should always be considered^3^.

Herein, we report a case report of an adult man with Weil’s disease causing multiorgan complications and highlight the management of acute pancreatitis and AKI in Weil’s disease. This case report is reported in line with the CAse REport (CARE) guidelines criteria^5^, which are used by authors, journal editors, and reviewers to increase the robustness and transparency of reporting cases.

## Case presentation

A 22-year-old male patient presented to the hospital with a chief complaint of persistent fever, nausea and vomiting (three times a day with an approximate volume of 100 ml), and jaundice for a week. The fever was continuous, causing headaches, and not accompanied by chills, sweating, and rash. Upper right abdominal pain that radiates to the back persisted for a week and worsened during the supine position and when eating. The pain reduced when sitting hunched over and with knees bent. Pain in the calf was also experienced by the patient. The patient felt a decrease in appetite and malaise. The patient experienced discoloration in urine and feces. There was no history of cough or cold in the last month. A history of consciousness disorders was absent. The patient and family had never experienced this complaint before. The patient denied any history of hypertension, diabetes, or drug and food allergies. Smoking and alcohol consumption were both denied. Two weeks ago, the patient’s residence had flooded. Diet from food stalls in the last month was absent. No history of traveling out of town.

The general health condition of the patient was weak, Glasgow Coma Scale score of E4V5M6, blood pressure of 130/80 mmHg, pulse rate of 100 beats per minute (bpm), respiratory rate of 20 bpm, the axillary temperature was 37.5°C, peripheral oxygen saturation was 98% room air. The patient’s body mass was 60 kg and height was 165 cm. The patient had conjunctival suffusion and icteric sclera. There were no signs of cyanosis, dyspnea, or enlargement of cervical lymph nodes. Lung and heart physical examinations were within normal limits. Abdominal examination showed a flexible abdomen (no distention), normal bowel sounds, liver and spleen were not palpable, and the pain was found in the left hypochondriac region and epigastrium. Cullen’s or Turner’s sign was absent. Extremities examination found tenderness in the right and left gastrocnemius muscle.

**Figure 1:**
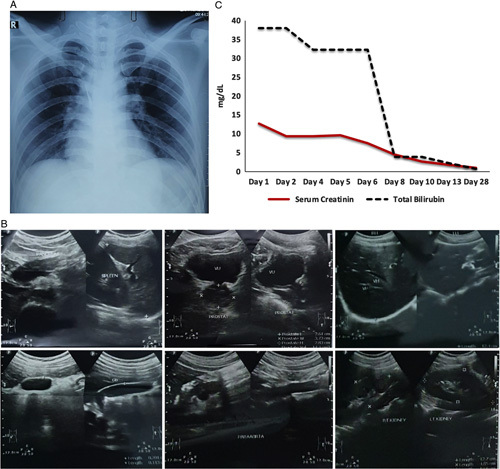
Chest radiograph examination showed: a thickening of the left hilum, and the heart abnormalities were absent (A). The results of the upper and lower abdominal ultrasound examination showed normal limits (B). Improvement of serum creatinine and total bilirubin levels of the patient (C).

Laboratory examination showed hemoglobin (Hb) of 12.2 g/dl, hematocrit (HCT) of 33.9%, mean corpuscular volume (MCV) of 80.3 fl, mean corpuscular hemoglobin (MCH) of 28.9 fl, leukocytes of 31.010/mm^3^, neutrophils of 91.7%, lymphocytes of 3.5%, platelets of 252 000/mm^3^, sodium (Na) level of 131 mmol/l, potassium (K) level of 6.7 mmol/l, chloride (Cl) level of 79 mmol/l, random blood glucose of 144 mg/dl, blood urea nitrogen (BUN) of 243 mg/dl, creatinine (Cr) of 12.66 mg/dl, albumin of 2.89 g/dl, alanine transaminase (ALT) of 385 U/l, aspartate transferase (AST) of 254 U/l, direct bilirubin of 28.03 mg/dl, total bilirubin of 38.03 mg/dl, partial thromboplastin time (PPT) of 16.5 s, and activated partial thromboplastin clotting time (APTT) of 27.4 s (Table 1). The patient tested negative for HBsAg, anti-HCV, and anti-HIV. Enzyme examinations showed amylase level was 556 U/l and lipase level was 3795 U/l. Blood gas analyses are presented in Table 1. Acidity (pH) of 7.33, partial pressure of carbon dioxide (pCO_2_) of 27, partial pressure of oxygen (pO_2_) of 105, bicarbonate (HCO_3_) of 14.2, base excess (BE) of −11.7, arterial oxygen saturation (SaO_2_) of 98%, and PaO_2_/FiO_2_ (P/F) ratio of 500. A complete urine examination showed cloudy yellow color, pH of 5.0, specific gravity of 1.015, protein of 1+, bilirubin of 3+, normal urobilinogen, erythrocytes of 3+, leukocytes of 1+, negative for ketone and nitrite, urine albumin-to-creatinine ratio (ACR) of 150, and protein–creatinine ratio (PCR) of 0.3. The coronavirus disease 2019 antigen and polymerase chain reaction swab results were all negative. The echocardiography examination showed normal limits. Chest radiograph examination suggested a thickening of the left hilum, and the heart abnormalities were absent (Fig. 1A). The results of the upper and lower abdominal ultrasound examination showed normal limits, as shown in Figure 1B. Based on the findings, the patient was diagnosed as having presumptive Weil’s disease with acute pancreatitis and AKI with hyperkalemia, hyponatremia, hypotonic hypovolemia, hypoalbuminemia, and metabolic acidosis. This diagnosis was made based on the 2012 WHO Faine modified score (based on six clinical criteria, two laboratory-defined criteria, and one epidemiological criterion)^6^. This patient had a modified Faine 2012 score of 35 (headache, fever, conjunctival fusion, muscle pain, particularly in the calves, jaundice, albuminuria/urea retention, and rainy season), indicating a presumptive diagnosis of leptospirosis. This diagnosis is supported by positive *Leptospira* IgM later during the follow-up. The patient had jaundice and severe renal dysfunction requiring hemodialysis; thus, the patient was diagnosed with severe leptospirosis or Weil’s disease.

**Table 1:** Laboratory parameters of the patient at hospital admission and during the follow-up.

		Follow-up day					
Laboratory parameter	Admission day	Day 3	Day 6	Day 8	Day 10	Day 13	Policlinic day 18
Hemoglobin, g/dl	12.2	9.4		9.4	7.2	7.8	12.6
Hematocrit, %	33.9	25.8		27.5	23.4	24.4	39.7
Mean corpuscular volume, fl	80.3	79.1		80.6	89.3	89.7	
Mean corpuscular hemoglobin, fl	28.9	28.8		27.6	27.5	28.7	
Leukocytes, mm^3^	31 010	20 600		33 610	19 790	11 220	9430
Neutrophils, %	91.7	88.5		90.8	85.1	71.9	71.3
Lymphocytes, %	3.5	3.9		2.2	6	9.8	16.1
Platelets, mmol/l	252 000	184 000		151 000	301 000	338 000	202 000
Sodium, mmol/l	131	137	133	128		145	142
Potassium, mmol/l	6.7	5.5	6.7	4.7		4.3	3.9
Chloride, mmol/l	79	94	91	98		107	106
Calcium, mg/dl		5.35	7.59	6.63	7.32		
Phosphate, mg/dl		9.49	13.17	7.01	4.88		
Blood gas analysis							
pH	7.33	7.37	7.25	7.35			
pCO_2_	27	27	28	34			
pO_2_	105	84	78	89			
HCO_3_	14.2	15.6	12.3	18.8			
Base excess	−11.7	−9.7	−14.9	−6.8			
SaO_2_, %	98	96	93	96			
PaO_2_/FiO_2_ ratio	500	400	371	424			
Procalcitonin, ng/ml		6.97		1672			
Random blood glucose, mg/dl	144					107	106
Blood urea nitrogen, mg/dl	243	189	199	113	65	38	5
Creatinine, mg/dl	12.66	9.36	9.65	4.51	2.64	1.79	0.93
Albumin, g/dl	2.89		2.73	3.0		3.13	3.9
Alanine transaminase, U/l	385			92		254	46
Aspartate transferase, U/l	254			58		56	25
Direct bilirubin, mg/dl	28.0		20.82	2.6		1.7	0.33
Total bilirubin, mg/dl	38.0	38.0	32.3	3.9	3.9	2.2	0.67
Amylase, U/l	556			417	194		
Lipase, U/l	3795			1416	633		

The patient was conditioned to fast and treated with an infusion of sodium chloride (NaCl) 0.9% at a volume of 2000 cm^3^ in 6 h, intravenous (i.v.) ceftriaxone at a dose of 1 g twice a day, i.v. metoclopramide at a dose of 10 mg three times a day, i.v. calcium gluconate at a dose of 1 g followed by dextrose 40% with insulin 2 IU for six times, avoided nephrotoxic drugs, and fluid balance I=O+500 ml. The consultation with the Nephrology Division suggested possible pseudohepatorenal syndrome, six times hyperkalemia correction, then a laboratory test was taken for post-correction random blood glucose and serum electrolytes, fluid maintenance with NaCl 0.9% : D10 = 2 : 2, insertion of a triple-lumen hemodialysis (HD) catheter/central venous catheter in the subclavian/jugular for parenteral nutrition, and central venous pressure was evaluated because of possible renal shutdown requiring hemodialysis. The patient then underwent hemodialysis due to refractory hyperkalemia with the following prescription: 2.5 h HD, 150 blood flow rate (QB), 500 dialysis fluid flow rate (QD), without heparin and ultrafiltration.

On the third day of hospitalization, abdominal pain was reduced, fever decreased, and the patient had no nausea and vomiting. Glasgow Coma Scale score was E4V5M6, blood pressure was 130/70 mmHg, pulse was 82 bpm, strong and regular pulse rhythm, respiratory rate of 20 bpm, axillary body temperature of 36.9°C, peripheral oxygen saturation of 98% at room air with urine production 3400 cm^3^ per 24 h. Blood culture, urine culture, procalcitonin, laboratory tests post hemodialysis, immunoglobulin (Ig) M and IgG *Leptospira* serology tests were performed. Laboratory examination results are presented in Table 1. Patient started enteral nutrition with Nephrisol at a dose of 100 cm^3^ six times a day over a nasogastric tube (and increased gradually), D10% infusion : NaCl 0.9%=2 :2, i.v. ceftriaxone at a dose of 1 gram twice a day, i.v. metoclopramide at a dose of 10 mg three times a day, i.v. calcium gluconate 1 ampoule given twice, paracetamol 500 mg three times a day over a nasogastric tube, calcium carbonate (CaCO_3_) dosed at 1 tablet three times, and lactulac syrup 15 ml three times a day.

On the sixth day, complaints of weakness and fever were gone, and the patient had stable vital signs with 2600 cm^3^ urine production for 24 h. The blood culture test and urine culture test showed no bacteria growth. Laboratory parameters are presented in Table 1. Potassium level was corrected, and electrolytes were examined post-correction, indicating Na 129 mmol/l, K 6.9 mmol/l, and Cl 91 mmol/l. The patient was planned to start a refined porridge diet, high in calories and sufficient protein, 2100 kcal per 24 h, infusion of 0.9% NaCl, rehydration 500 cm^3^ for an hour, followed by 1500 cm^3^ per 24 h over CVC. Sodium bicarbonate 75 mEq in NaCl 0.9% 500 cm^3^ (drip) over the first line of i.v., the second line was D10%+insulin of 10 IU, and other therapies remained the same.

Eight days after hospitalization, the patient’s condition was still weak, but there were improvements in appetite, and the patient was able to defecate. Vital signs were normal and urine production of 3000 cm^3^ per 24 h. Laboratory examinations are presented in Table 1. Enzyme examination showed amylase levels of 417 U/l and lipase levels of 1416 U/l. The sodium bicarbonate drip was stopped and replaced with oral sodium bicarbonate. The patient underwent a high-calorie coarse porridge diet with enough protein of 2100 kcal per day. The patient was transferred to a low-care room. The changes in serum creatinine and total bilirubin levels of the patient are presented in Figure 1C. On day 9, the *Leptospira* IgM serology was positive.

On day 10, the patient had no complaints. The laboratory parameters gradually returned to normal (Table 1). Enzyme examination showed amylase levels of 194 U/l and lipase levels of 633 U/l. *Leptospira* IgM serology was positive, while and *Leptospira* IgG yielded negative. Infusion therapy was NaCl 0.9% 1000 cm^3^ : D5% 1000 cm^3^ per 24 h, while other therapies remained the same. On day 13, the patient had no complaints and had normal vital signs. Laboratory examination results suggested a good progression of the disease (Table 1). The patient was discharged from the hospital. An outpatient visit on the day 18 indicated a good recovery and the laboratory test results are presented in Table 1.

## Discussion

We reported a rare case of Weil’s disease with acute pancreatitis and AKI. This case is unique because the patient also has hyperkalemia, hyponatremia, hypoalbuminemia, hypotonic hypovolemia, and metabolic acidosis. Since Weil’s disease has the potential to be fulminant and fatal, the success of the management of this case needs to be discussed. This patient showed common symptoms of leptospirosis; the most common symptoms include fever, myalgia, headache, jaundice, hepatomegaly, splenomegaly, and renal involvement. The diagnosis in this patient was made using the 2012 WHO Faine modified score, where the patient had a score of 35, indicating a presumptive diagnosis of leptospirosis. The patient had jaundice and severe renal dysfunction requiring hemodialysis; thus, the patient was diagnosed with severe leptospirosis or Weil’s disease.

This patient had symptoms of sudden epigastric pain, pain radiating to the back, anorexia, nausea, decreased bowel sounds, fever, jaundice, and hematemesis. Based on the consensus of acute pancreatitis in Indonesia, there were two of three criteria, namely typical abdominal pain and an increase in serum amylase and lipase levels more than three times the upper limit of normal, without radiological findings. There were no other causes of pancreatitis found in this patient, such as gallstones, alcoholism, and drug use.

Infectious diseases-inducing sepsis are still a significant problem in Indonesia^7^, and early diagnosis and effective antibiotic treatment are required to avoid the high mortality rate. Penicillin 1.5 million units i.v. per 6 h, ampicillin 0.5–1 g i.v. per 6 h, ceftriaxone 1 g i.v. per 24 h, or cefotaxime 1 g i.v. per 6 h are the antibiotics used to treat severe leptospirosis in hospitals^8^. Antibiotics in patients with severe acute pancreatitis are only given for extrapancreatic infections such as cholangitis, infections due to catheter insertion, bacteremia, urinary tract infections, and pneumonia. Antibiotics that have been widely studied and can penetrate the pancreas are carbapenem, quinolones, metronidazole, and high-dose cephalosporins^9^. A recent study found that the most sensitive antibiotics to Gram-negative bacteria in Dr Soetomo General Academic Hospital are amikacin, cefoperazone, and piperacillin-tazobactam^10^. This patient was given i.v. ceftriaxone 1 g per 12 h for 7 days. The results of blood and urine cultures did not reveal any microbes. Antibiotics were continued for up to 10 days.

This patient was given initial rehydration using 2000 ml isotonic NaCl 0.9% crystalloid solution in the first 6 h, then given maintenance fluid dextrose 10% 1000 cm^3^ and NaCl 0.9% 1000 cm^3^ in 24 h using central venous access. Early aggressive i.v. fluid resuscitation can prevent pancreatic necrosis by maintaining macrocirculation and microcirculation^11^. Aggressive hydration (250–500 ml per hour) of isotonic crystalloid fluids should be given unless cardiovascular and/or renal comorbidities are present. In addition, the combined effect of microangiopathic and pancreatic edema decreases blood flow, leading to increased cell death, necrosis, and sustained release of pancreatic enzymes that activate multiple cascades. Inflammation also increases vascular permeability, causes increased third-space fluid loss, and worsens pancreatic perfusion leading to pancreatic parenchymal necrosis and cell death^9^,^12^.

In severe acute pancreatitis, total parenteral nutrition should be avoided, but integrated partial parenteral nutrition should be considered to meet caloric and protein requirements if the enteral route is not available, not fully tolerated, or there are contraindications to the enteral route (bowel obstruction, prolonged paralytic ileus, mesenteric ischemia)^9^,^12^. This patient was given integrated partial parenteral nutrition: temporary fasting in the first 24 h because of nausea and abdominal pain, then a nasogastric tube was inserted to enter 100 cm^3^ of Nephrisol per 4 h, then increased gradually to 200 cm^3^ per 4 h. On the fifth day, the nasogastric tube was removed, and started a low-calorie, high-protein diet of refined porridge with adequate protein of 2100 kcal per 24 h. In addition to enteral nutrition, i.v. parenteral nutrition was also given with infusion, consisting of 10% dextrose 1000 cm^3^ with 0.9% NaCl 1000 cm^3^ per 24h using central venous access.

Renal involvement is common in leptospirosis and its manifestations vary from mild proteinuria and changes in urine sediment to severe renal failure^13^. Two mechanisms have been suggested in the development of kidney failure in leptospirosis: direct nephrotoxic due to several endotoxins or endotoxin-like substances, and the anoxic effect due to kidney circulation disturbances^14^. There was no oliguria in this patient. Laboratory examination showed increased levels of BUN and serum creatinine, hyperkalemia, and metabolic acidosis. The main histologic findings are acute interstitial nephritis and acute tubular necrosis. Leptospirosis-induced AKI is usually nonoliguric and hypokalemic^15^. Hyperkalemia reflects extensive cellular damage, resulting in massive potassium leakage into the systemic circulation^15^. Our patient had fluid rehydration and correction of hyperkalemia 12 times, but still refractory hyperkalemia was still found, resulting in the patient undergoing early hemodialysis. After hemodialysis, fluid administration was carried out with close monitoring, and found decreased levels of BUN, serum creatinine, electrolyte, also blood gas improvements were found. Although there is no consensus on the best dialysis modality for leptospirosis, early dialysis could reduce mortality^15^. Normalization of serum urea and creatinine levels usually occurs in the second week of illness, along with an increase in platelet count and a decrease in bilirubin levels^15^, and this is also evident in our patient.

This case report has strengths and limitations that need to be discussed. This is unique because Weil’s disease with acute pancreatitis and AKI is rare, and the patient was managed successfully with aggressive treatment. The diagnosis of Weil’s disease was made based on the 2012 WHO Faine modified score. Serologic tests, such as the microscopic agglutination test and enzyme-linked immunosorbent assay for leptospirosis, were not carried out on the patient due to the unavailability of the tests.

## Conclusion

In Weil’s disease, with complications of acute pancreatitis and AKI, aggressive and proper treatments with appropriate antibiotics are needed. Early hemodialysis would also benefit the patient if the indicators of AKI are present.

## Ethical approval

The study is exempt from ethical approval in our institution.

## Patient consent

Written informed consent was obtained from the patient for the publication of this case report and accompanying images. A copy of the written consent is available for review by the Editor-in-Chief of this journal on request.

## Sources of funding

This study has received no external funding.

## Author contribution

R.B.H.: clinical management, patient care, and manuscript preparation and editing. B.B., M.R., M.V.A., and U.H.: patient care and manuscript editing.

## Conflicts of interest disclosure

The authors declare no conflicts of interest.

## Research registration unique identifying number (UIN)

None.

## Guarantor

Bramantono Bramantono.

## Provenance and peer review

Not commissioned, externally peer-reviewed.
